# LIMPRINT: A Sociological Perspective on “Chronic Edema”

**DOI:** 10.1089/lrb.2018.0082

**Published:** 2019-04-22

**Authors:** Stuart Nairn, Eleanor Dring, Aimee Aubeeluck, Isabelle Quéré, Christine Moffatt

**Affiliations:** ^1^ School of Health Sciences, University of Nottingham, Royal Derby Hospital Center, Derby, United Kingdom.; ^2^ Nottingham University Business School, University of Nottingham, Jubilee Campus Nottingham, United Kingdom.; ^3^ School of Health Sciences, University of Nottingham, Queens Medical Center, Nottingham, United Kingdom.; ^4^ Montpellier Medecine Vasculaire, EA2992, Universite Montpellier I, CHU Saint Eloi, Montpellier, France.; ^5^ School of Social Sciences, Nottingham Trent University, Nottingham, United Kingdom.

**Keywords:** chronic edema, sociology, Pierre Bourdieu (social capital), lymphedema, lymphoedema

## Abstract

***Background:*** Chronic edema is a condition that is biologically complex, distressing for patients and sociopolitically weak. Like many other complex and chronic conditions, it has a low status within health care. The result is that it has a low priority in health policy and consequently is undervalued and undertreated. While evidence-based practice promotes a hierarchy of evidence, it is also the case that clinical practice is influenced by a hierarchy of social status. These are as much political as they are scientific.

***Methods and Results:*** This article will provide an explanation for why chronic edema is a low priority. It will do this through a critical review of the literature. We examine this through the theoretical lens of Pierre Bourdieu. The sociology of Bourdieu frames an understanding of power relations through habitus, field, and capital. We will employ these theoretical tools to understand the way that chronic edema is situated within the policy arena. We identify a number of social mechanisms that affect the status of chronic edema, including diagnostic uncertainty, social capital, scientific capital, cultural capital and economic capital.

***Conclusion:*** We argue that a whole system approach to care, based on human need rather than unequal power relations, is a prerequisite for the delivery of good health care. The specialty of chronic edema is not a powerless group and we identify some of the ways that the social mechanism that acts as barriers to change, can also be employed to challenge them.

## Introduction

Health care systems confront many pressures. These pressures significantly affect the type of care delivered to patients. While the basic principles of health care are to maximize health and minimize illness, the application of policy takes place in complex contexts. An approach based on social justice would endeavor to deliver services based on fairness and human need. However, decisions about the provision of care is affected by a variety of social, economic, cultural, and political factors.^1^ Any analysis of policy needs to engage with these different sociopolitical factors so that evidence-based policy can be applied based, not only on identified needs, but also an approach that recognizes the broader context. In this article, we will explore this context as it effects chronic edema, which is underrecognized and undertreated.^2,3^

We will approach this through the theoretical lens of the sociologist Pierre Bourdieu. Bourdieu identified fundamental aspects to the social world, habitus, capital, and field.^4–6^ Habitus is about how social behavior is embedded in social action. Behavior is therefore a set of dispositions that are durable, often regulated that are not straightforwardly consciously enacted, but internalized and embodied in human praxis. Habitus is not simply rule following, but a disposition to adapt one's actions to the social fields one inhabits.

Fields are socially structured spaces that incorporate practice rules. The field creates regulatory practices and shapes the positions people occupy within an institution, an organization, and can be potentially referred to as a set of objectively defined social locations. Ones position within it are products of past actions and the field and are a focus of continuous struggle and conflict between different groups both within a field and alongside other models of health care and the inherent tension these engender. So, for the purposes of this article, we are interested in the field of health care within which there are subfields of medicine and allied health professionals and within that further specialties with their own agendas. The picture is complex with a frequent divide between private and public health care systems. Attempting to promote a particular part of the field (e.g., chronic edema) entails a struggle for influence within the broader field of health care and this is not simply a polite discussion and dialog based on rationally generated evidence-based knowledge, but a competitive engagement with deeply embedded social structures.

This engagement with the field involves the use of power. Power is utilized through the third component of Bourdieu's theory of capital. Capital is not simply economic, but also symbolic. There are multiple types of capital but the central idea of capital is that people and groups access capital to different degrees that enable them to have influence and power within a field of practice. So academic credentials provide a person more power in an educational field. Throughout this article we will be referring to different types of capital, including scientific, social, cultural, and economic capital as ways that chronic edema is marginalized within the health care field.

We should note that while we have identified these three concepts of habitus, field, and capital as discrete ideas, Bourdieu used these ideas as inextricably linked in the real world. It has been noted for example that, in the health care literature, the idea of capital has been emphasized at the expense of field, which potentially results in a culturalist explanation. The consequence is an insufficient account of the way that capital is only useful if it is situated within a broader structuralist paradigm.^7,8^ However, field provides a key component of an explanation for health service delivery that is focused on the way that existing structures of domination of the social relations that exist within that field and how the field have been formed and could potentially be changed by the social forces within that field. Within this article, we will integrate these ideas as an explanatory means to understand the undervalued nature of chronic edema in the health care field.

## The Problem of Diagnosis

Diagnosis is a process whereby disease is distinguished from health, which should then lead to an appropriate treatment.^9^ Problems may arise when there is uncertainty about how to define symptoms. But from a sociological perspective, diagnosis is also a social process in which medicine identifies what is significant in the diagnostic process.^10^ In short, diagnosis frames the experience of illness, providing it with a label and decides which experiences of illness are privileged and which are silenced.^11^ Therefore, diagnosis is not just a matter of biological dysfunction but exists in a social and political context. Furthermore, the application of a diagnosis transforms the individual's sense of himself or herself as they generate a new narrative based on this label. While sociologists have been concerned to broaden the scope of diagnosis, incorporating lay experiences into a relational approach to managing illness; clinicians focus and appreciate the value of a precise definition. This can be seen clearly in a debate in the *Journal of Lymphedema*.^12^ This discussion was concerned to identify whether the term lymphedema or chronic edema should be used.

The complexity of this discussion is not our concern within this article, but what these clinicians were concerned about was the policy implications of the two terms. While there seemed to be a general preference for the term chronic edema, there was also anxiety that this would be confusing for managers providing the service who are familiar with the term lymphedema but not chronic edema. Perhaps they could be used synonymously despite the different diagnostic and treatment regimens associated with the different definitions? Would a change in name affect funding and service provision? The authors are clearly concerned here with the tension between diagnostic accuracy and the profile of the condition, which they are professionally engaged with. The language of diagnosis clearly matters in relation to both treatment and the profile of the disease and the existence of this debate clearly illustrates the ongoing problems in providing a clear focus in the policy arena.

The issue of diagnosis is a profoundly complex issue within the field of chronic edema and lymphedema. The debate rages over whether chronic edema and lymphedema are purely a set of symptoms or whether they constitute a newly emerging health care problem. This is deeply embedded in the newly emerging physiological data that challenges long-held beliefs about the function of the venous and lymphatic circulation and is deeply engaging academics in this field. However, this debate is not influencing clinicians who work in nonspecialist areas of practice.

The heterogeneous nature of the patients affected leads to a lack of ownership of the problem. In some situations, such as following cancer treatment, lymphedema may be perceived by clinicians as a minor irritation to a patient rather than having a profound effect on people's lives. Clinicians may actively distance themselves from the implications of their intervention rationalizing that treatment of the underlying disorder is the only relevant factor.

The lack of clarity over the definitions used to define chronic edema and lymphedema are leading to confusion over the objectives of treatment and what constitutes an effective outcome. Clinical outcome measures, such as volume change in a limb, have some legitimacy and understanding in a specialist lymphedema audience. There is, however, no agreement over the size of volume change required to be effective or a link to issues, such as health-related quality of life, function, and cost-effectiveness. The term “volume change” is not understood in the wider audience of health care, in particular reimbursement and health care agencies responsible for provision of care.

The patient understanding of the relevance of volume change as an outcome measure has received scant attention and issues such fear of cellulitis and unpleasant symptoms are likely to be of greater importance. The lack of a common language that allows a comparison of chronic edema with other chronic illnesses further reduces the potential for a high social capital and influence on the health care systems.

## Cultural Capital and the Prestige of Diseases

Having a firm, clearly defined diagnosis and attaching that to a specialist practice alongside a program of research, all contribute to the status of a disease. Some diseases are acknowledged to be low status and neglected. For example, there is now an extensive literature on neglected tropical diseases.^13^ Scientific capital directs researchers to those areas, where there is the greater prestige, those that are considered more important. As Bourdieu^14^ points out there is a hierarchy of science entailing a struggle for funding that generates a market approach to knowledge. The particular position of a group within the field of health is dependent on the historical crystallization or previous struggles and the way that various forms of symbolic capital have become embedded in the field. Chronic edema has struggled to gain attention for a number of reasons.

There is good evidence that chronic edema is extensive.^2,15^ However, this is not reflected in its profile as an area of concern. But more fundamentally this lack of interest reflects broader ideas about the prestige of diseases and specialties. In short, some diseases have more prestige than others do. In general, the acute conditions that respond to invasive procedures, vital organs in the upper part of the body have higher prestige than chronic conditions that affect the lower body or nonspecific parts of the body.^16,17^ There is also evidence that medical students who are more career oriented tend toward technique-oriented specialties that reflect biomedical practices and are seen to have higher prestige.^18^ Chronic edema is not mentioned in any of these research articles, but given the chronic nature of the illness and the treatment regimens that are primarily nurse related and nonsurgical, it is reasonable to suggest that chronic edema has low prestige or what Bourdieu would refer to as cultural capital.

## Scientific Capital

To examine science and scientific practice, sociologists explore what social mechanisms are in place that orient science to particular objects of knowledge and not others. Why is it that some subjects have higher status than others and how do they accumulate scientific capital.^19^ As we have already argued, different medical specialties have higher prestige than others and it is clear that chronic edema is a specialty that struggles to gain attention. The efficacy of treatment regimes is only partly a factor in this struggle for attention. So for example, the efficacy and usefulness of genetic knowledge continues to be modest, but has managed to attract high levels of investment due to its status as “big science.”^20^ Some have noted how attention for funding is rooted in what they refer to as expectational capital^21^ in which researchers are able to promote their area through promises of future success that are based on inflated claims about the possibilities of success rather than a realistic evaluation. However, researchers are simply working within the paradigm of funding streams and unlikely to access support if they do not put an optimistic gloss on their projects. Working in the field of chronic edema is unlikely to generate this kind of interest, as it does not conform to an ideology of clear-cut interventions.

## Cultural Capital and Media Representations

The evolution of a specialty depends upon a variety of factors, the type of condition, its scientific status, the organizational possibilities, the usefulness in meeting health care and institutional priorities, the prestige associated with the problem, and which social groups suffer from it. But there is also a less tangible but nonetheless important cultural context in which health care is delivered. The media is important in the way that it represents health and health care systems.^22^ It can even influence the development of a specialty. Emergency care has a long history of being a medical backwater with a low status. The open-ended nature of its access (so anyone can turn up), and its subsequent lack of focus on a specific condition and its history as little more than an add on to orthopedics has resulted in it being defined as a low priority.^23^ Many factors have influenced this change, not least it focuses on the acute and traumatic, but part of this has been its cultural representations, which have found their place in a cultural context in which the emergency, the heroic, the world weary, and the nonconformist individual operates in a world that is anxious and fragile.^24^ The high profile of these television programs, both fictional and documentary, has contributed to profile of the specialty and is acknowledged by leading figures in the specialty itself.^24^

Specific diseases are also promoted in ways that reflect a strong cultural component. For example, Lyme disease has had its profile substantially enhanced using celebrity support.^25^ Media depictions can also have negative effects. In an analysis of the program, Seinfeld, negative and stigmatizing representations of skin conditions were the norm and the subject of satire, ridicule, and exaggeration.^26^ So, problems of the skin are less likely to engender a sympathetic representation. It lacks drama and feeds into a general cultural discourse of disgust that is often internalized by patients.^27^

## Economic and Social Capital

To examine the health care sector as a field is to understand the competing nature of different actors within a social system who employ different strategies to enhance the position of their speciality.^7^ Chronic edema has problems asserting itself as condition worthy of attention by policy makers. Chronic conditions are often spatially spread through the community rather than organized in a defined space within a hospital. For example, surgical conditions generate teams who focus on defined problems, whereas chronic edema is often situated in the home and nursing homes, often unnoticed and undertreated.^2^

It is, however, possible to make an economic case for chronic edema. If the idea of chronic edema is broadened, then an argument can be made to support more attention. There is literature that identifies the effects of living with chronic edema. So, living with chronic edema generates profound psychosocial consequences, including emotional issues, body image, an undermining of self-confidence and a sense of self, a feeling of being unattractive and disgusting, depression, anxiety, and psychological distress. It can affect sexuality, relationships, and social contacts, which can lead to social isolation and a sense of abandonment from health care system that provide minimal support.^27^ While these aspects should promote greater attention based on human need, these issues tend to have low social capital and prestige when faced with more clearly identified medical problems with clear medical solutions.^17^

Furthermore, the costs of living with chronic edema is dependent on service provision. Boyages et al.^28^ have identified significant financial costs borne by Australian women with lymphedema that is secondary to breast cancer as they must pay for treatment products. Wagner et al.^29^ have identified significant problems associated with accessing insurance cover in Japan. Weiss^30^ has noted that in the USA there are similar problems arguing that despite the low cost of treatments, and the potential savings in prevention with complications, such as cellulitis, insurance companies have actively resisted any attempts to fund these treatments. Identifying the costs of chronic edema is always problematic but it is reasonable to include social factors in any costing process. These might include the increasing care burden on families, the increased levels of sickness, loss of productivity, and quality-of-life issues such as mental health services. Humphreys and Thomas^31^ argue that there is ultimately a cost saving to be made if all these factors can be considered. Gutknecht et al.^32^ support this by incorporating direct and indirect costs into their analysis and suggest that a comprehensive treatment program improves care and also has the potential to decrease costs. However, these articles are cautious about cost savings and therefore have limitations in promoting and setting up such programs of care in the competitive environment of health care systems.

## Conclusion and Implications

**FIG. 1.**
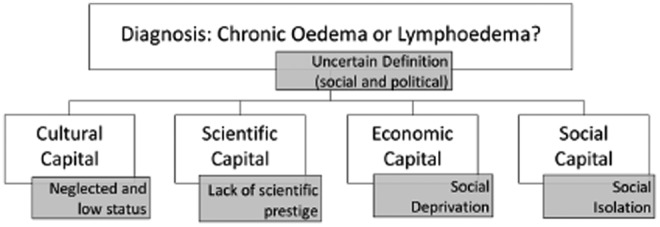
A sociological perspective on “Chronic Edema.”

According to Bourdieu^19^ the obstacles to scientific progress are fundamentally social. Science is inevitably a part of the political domain. However, it should progress in a way that is not reducible to politics. The purpose of sociology is not to relativize science but to situate it and then to understand what social mechanisms are at work in defining the practice of science. Bourdieu situates scientific knowledge within a field, in which struggles take place for influence based upon their position within that field and the amount of capital that agents can employ within that struggle. Health care is not just about science however, but is a clinical practice employed on people and it is the purpose of health care to deliver care that meets the needs of people. While the idea of what a need is may be problematic to define, the argument that these needs are not being met for people with chronic edema is compelling. We have outlined some of the key issues affecting this group (Fig. 1).
